# Management of type 1 gastric neuroendocrine tumors: an 11-year retrospective single-center study

**DOI:** 10.1186/s12876-023-03079-6

**Published:** 2023-12-14

**Authors:** Ying-Ying Chen, Wen-Juan Guo, Yan-Fen Shi, Fei Su, Fu-Huan Yu, Ru-Ao Chen, Chao Wang, Ji-Xi Liu, Jie Luo, Huang-Ying Tan

**Affiliations:** 1 Beijing University of Chinese Medicine, 11 North Third Ring East Road, Beijing, 100029 China; 2 Department of Integrative Oncology, China-Japan Friendship Hospital, No. 2 Yinghuadong Street, Beijing, 100029 China; 3 Department of Gastroenterology, China-Japan Friendship Hospital, No. 2 Yinghuadong Street, Beijing, 100029 China; 4 Department of Pathology, China-Japan Friendship Hospital, No. 2 Yinghuadong Street, Beijing, 100029 China; 5 Digestive Disease Center, Beijing United Family Hospital, No. 2 Jiangtai Road, Beijing, 100015 China

**Keywords:** Type 1 gastric neuroendocrine tumors, Management, Recurrence, Prognosis

## Abstract

**Background:**

Type 1 gastric neuroendocrine tumors (NETs) are relatively rare to the extent that some physicians have little experience in diagnosing and treating them. The purpose of this study was to increase the understanding of the disease by analyzing and summarizing the management and prognoses of patients with type 1 gastric NETs at our center.

**Methods:**

The data of 229 patients (59.4% female) with type 1 gastric NETs who were treated at our center during 2011–2022 were retrospectively analyzed.

**Results:**

The average patient age was 50.5 ± 10.8 years. Multiple tumors affected 72.5% of the patients; 66.4% of the tumors were < 1 cm, 69.4% were NET G1, and 2.2% were stage III-IV. A total of 76.9% of the patients had received endoscopic management, 60.7% had received traditional Chinese medicine treatment, 10.5% received somatostatin analogues treatment, and 6.6% underwent surgical resection. Seventy patients (41.2%) experienced the first recurrence after a median follow-up of 31 months (range: 2-122 months), and the median recurrence-free time was 43 months. The 1-, 2-, and 3-year cumulative recurrence-free survival rates were 71.8%, 56.8%, and 50.3%, respectively. During a median follow-up of 39 months (range: 2-132 months), one patient had bilateral pulmonary metastasis, and no disease-related deaths were observed.

**Conclusion:**

Type 1 gastric NETs have a high recurrence rate and a long disease course, underscoring the importance of long-term and comprehensive management.

**Supplementary Information:**

The online version contains supplementary material available at 10.1186/s12876-023-03079-6.

## Introduction

Gastric neuroendocrine tumors (NETs) are a type of tumor with strong heterogeneity, and their incidence has been increasing over the past few decades [1]. Data from the Surveillance, Epidemiology, and End Results Program (SEER) database of the US National Cancer Institute suggest a 15-fold increase in the incidence of gastric NETs from 1973 to 2012 [2]. Well-differentiated gastric NETs are classified into three main types based on their pathogenesis [3], each exhibiting distinct biological behavior, prognosis and management [4, 5]. Accurate classification is therefore essential. Type 1 gastric NETs are the most common, accounting for approximately 80–90% of cases. They are associated with hypergastrinemia and autoimmune gastritis (AIG) [6, 7]. Recurrence is relatively common [8], necessitating the correct identification of type 1 gastric NETs and long-term follow-up. However, due to the rarity, heterogeneity and complexity of gastric NETs, there are few large-sample-size and long-term follow-up studies available to provide an overview of the clinicopathological characteristics, treatment outcomes and prognoses of type 1 gastric NETs. Thus, accurate classification and long-term management of type 1 gastric NETs present considerable challenges. In this study, we sought to perform a comprehensive retrospective analysis of our recent 11-year experience with type 1 gastric NETs at our neuroendocrine tumor center, aiming to provide more robust evidence to aid clinicians in making diagnostic and treatment decisions.

## Materials and methods

### Patient selection

This was a single-center retrospective analysis of type 1 gastric NETs evaluated or treated at the China-Japan Friendship Hospital from November 2011 to October 2022, and only patients with definitive histopathological and biochemical AIG-related type 1 gastric NET diagnoses were included in the study. This study was approved by the Clinical Research Ethical Committee of China-Japan Friendship Hospital (ethics approval number: 2022-KY-097-2). The study was conducted according to the Declaration of Helsinki, and full informed consent for data collection was obtained from all the included patients.

### Diagnostic criteria

The pathological grading of all patients was based on the 2019 World Health Organization (WHO) classification and grading criteria for neuroendocrine neoplasms (NENs) of the GI tract and hepatopancreatobiliary organs [9]. Clinical staging was determined according to the American Joint Committee on Cancer (AJCC) eighth edition of TNM staging [10] and TNM staging from NCCN guidelines for NETs of the stomach [11].All pathological grades and clinical stages were re-evaluated by senior physicians according to the above standards. Clinical classification was determined according to the classification characteristics of gastric NETs in digestive system tumors in the 2019 WHO classification [3]. Diagnostic criteria for type 1 gastric NETs were as follows: (1) well-differentiated gastric NETs proved by biopsy or surgery with histopathological confirmation, with background gastric mucosal atrophy in the nontumor area, accompanied by a series of neuroendocrine cell hyperplasia; (2) features consistent with the characteristics of AIG, such as the presence of positive anti-parietal cell antibodies (PCA) or positive anti-intrinsic factor antibodies (IFA), vitamin B12 deficiency, and a reduction in inherent glands; (3) achlorhydria; (4) hypergastrinemia; and (5) a diagnosis of type 1 gastric NETs by the researchers based on a comprehensive analysis of a combination of the clinical symptoms, endoscopic features, pathological morphological characteristics, and laboratory findings.

### Data collection and follow-up

Data for patients with type 1 gastric NETs were collected retrospectively, including patient demographics, clinical symptoms, tumor characteristics, histological results, treatment and follow-up. We conducted a retrospective analysis of the patients mainly by consulting patient diagnosis and treatment information through outpatient and inpatient electronic medical record information systems and telephone follow-up with patients. The deadline for follow-up was October 31, 2022.

A criterion for recurrence was established by experts in the Department of Pathology, Gastroenterology, and Oncology who were on the team at our NET center based on the ENETS guidelines and clinical experience. Type 1 gastric NET recurrence was defined in the following manner.

Endoscopic appearance (Table 1) combined with biopsy pathological results allowed cases to be allocated to categories as follows:

1. For patients who had undergone complete resection, once a new lesion (i.e., the number of polyps and/or the maximum diameter increased) was found and confirmed to be a gastric NET by histopathology, we judged that the tumor had relapsed;
2. For patients who had undergone incomplete resection (resection of only larger lesions), if the number of polyps and/or the maximum diameter increased (such as an increase from Level A to Level B) and gastric NETs were confirmed by histopathology, we judged that the tumor had relapsed.

**Table 1:** Evaluation of gastroscopic manifestations of type 1 gastric NETs

Endoscopic manifestation	Level 0	Level A	Level B	Level C	Level D
Number	0	1–3	4–6	7–10	> 10
Diameter	0 cm	Granular shape	< 0.5 cm	≥ 0.5 cm, < 1 cm	≥ 1 cm

### Statistical analysis

All statistical analyses (frequencies, descriptive statistics, Kaplan-Meyer curves, log‐rank tests and Cox proportional hazards regression model assessment) were performed with the SPSS 26.0 software package (IBM SPSS Statistics). Differences were considered statistically significant when the *P* value was < 0.05. Categorical variables were expressed as frequencies and rates, and continuous variables were expressed as the mean ± SD or median (range or 25-75th interquartile range, IQR). Recurrence‐free survival (RFS) was analyzed using Kaplan‐Meier methods. Log‐rank testing and the Cox proportional hazards regression model were used to determine whether there was a difference between RFS in type 1 gastric NET patients stratified by different clinicopathological and treatment strategies.

## Results

### Clinicopathological features

#### Patient demographics and initial symptoms

The clinicopathological data of 229 patients with type 1 gastric NETs were collected from November 2011 to October 2022 in this study; 93 males and 136 females were included, and the average age of the patients was 50.5 ± 10.8 years (range: 21–82 years).

One hundred and sixty of the 229 patients visited the doctor due to symptoms, among which the most common manifestations were abdominal distension (59 cases), abdominal pain (46 cases), belching (21 cases), postprandial fullness (20 cases), and dizziness (18 cases); 38 patients were asymptomatic, and gastric NETs were detected incidentally through gastroscopy during a medical examination.

#### Endoscopic manifestations

**Fig. 1:**
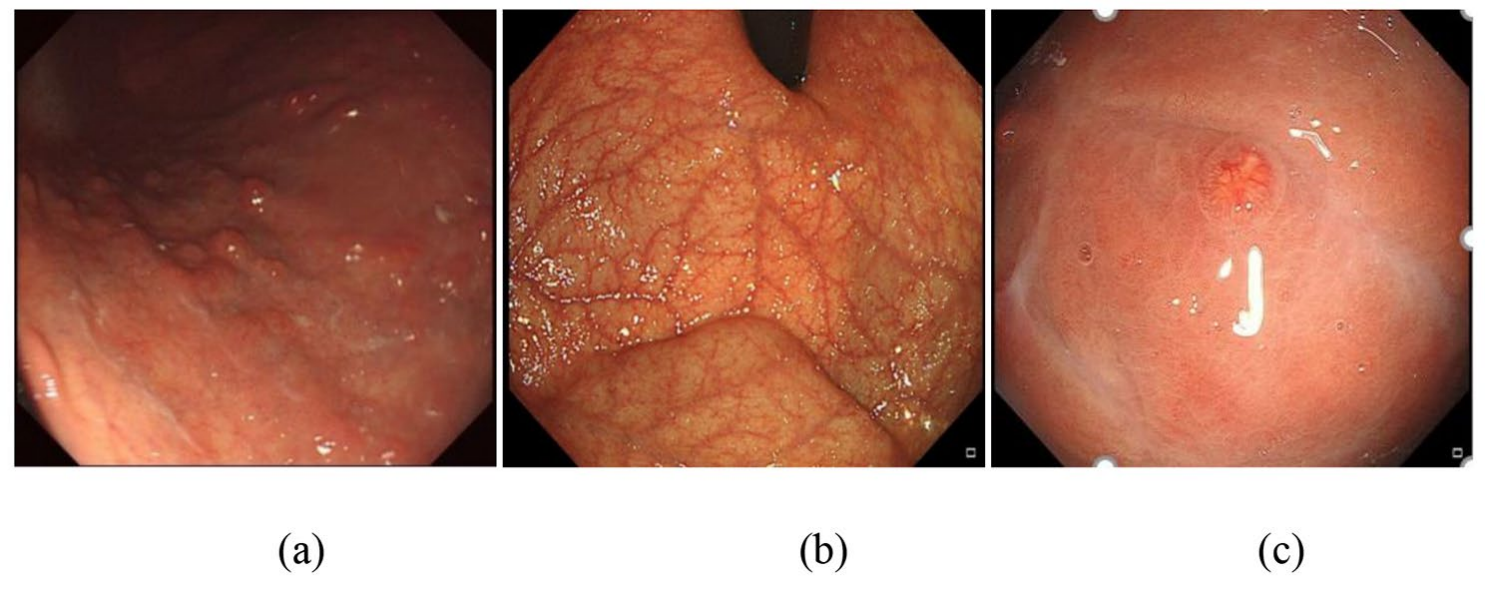
Endoscopic images in type 1 gastric NET patients with autoimmune gastritis (AIG). (**a**) NETs are minute (less than 0.5 cm) and frequently present as multiple reddish polypoid lesions. (**b**) Marked vascular visibility and the disappearance of folds without an atrophic border were observed. (**c**) Close-up observation of a sessile polyp; the tumor is gently elevated, and its surface is red with an enlarged gastric pit and dilated vessels

Among the 229 patients, 166 patients had multiple tumors, and 47 patients had single tumors. Most of the detected tumors (n = 152; 66.4%) were found to be < 1 cm, whereas 35 (15.3%) were 1 to 1.9 cm, and 9 (3.9%) were ≥ 2 cm. The tumors were located in the gastric body (n = 116), fundus (n = 20), and fundus and body (n = 78) of the stomach. The tumors of 197 patients were polypoid, 12 patients had polyps with erosion, 6 patients had erosion or ulcer, and 5 patients had coarse granular lesions. Endoscopic ultrasonography was performed in 85 patients, and 39 patients (45.9%) had tumors located in the mucosa, 45 (52.9%) in the submucosa, and 1 (1.2%) in the muscularis propria. A total of 168 patients (73.4%) showed evidence of atrophic gastritis (marked vascular visibility and atrophy of the gastric mucosa were observed), and 9 patients (3.9%) showed evidence of nonatrophic gastritis. Typical endoscopic images of gastric NETs with AIG are shown in Fig. 1.

#### Histopathological characteristics

**Fig. 2:**
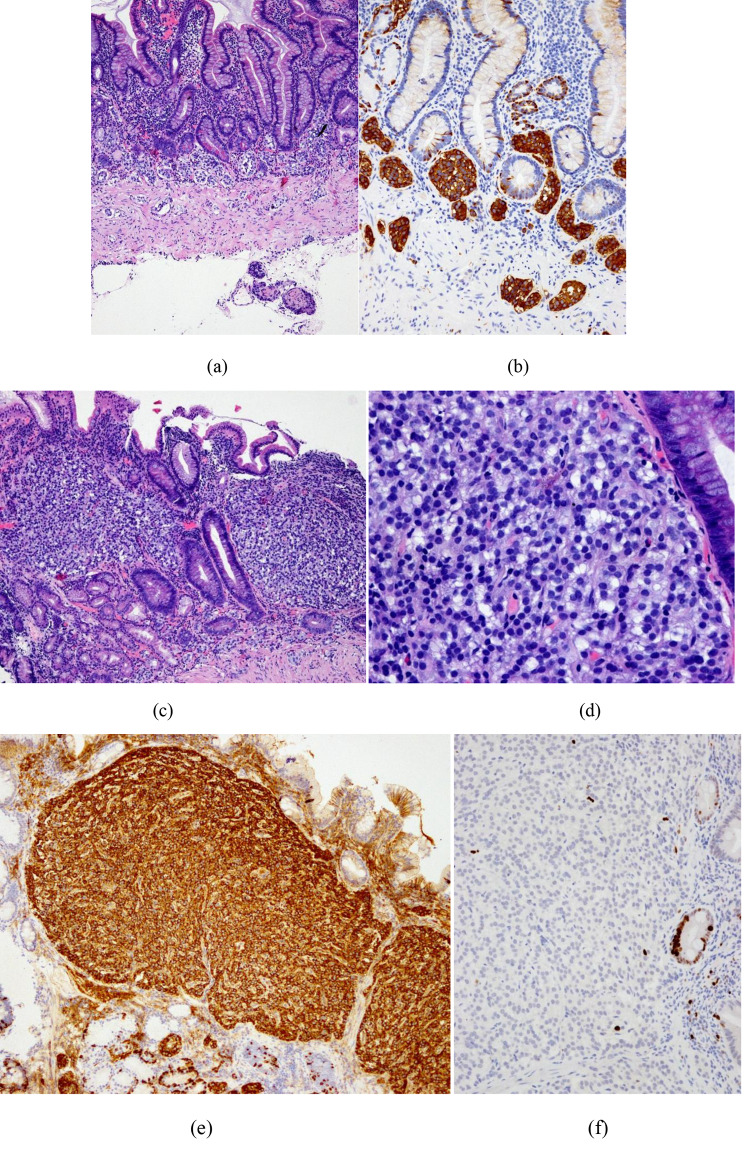
Typical histopathological findings of AIG-related type 1 gastric NETs. (**a**) Severe atrophy of the inherent glands in the gastric mucosa accompanied by intestinal metaplasia and muscularis mucosa hyperplasia, with neuroendocrine cell hyperplasia visible in the base of the gastric mucosa and muscularis mucosa (hematoxylin and eosin staining, 100×); (**b**) Linear and micronodule hyperplasia of neuroendocrine cells are observed at the base of the gastric mucosa and muscularis mucosa (Syn immunostaining, 200×); (**c**) A NET with background gastric mucosal atrophy and the presence of nodular hyperplasia of neuroendocrine cells (hematoxylin and eosin staining, 100×); (**d**) A NET, rich in cells and a blood supply and uniform in size, with nuclei showing “salt and pepper” chromatin and rare mitotic activity (hematoxylin and eosin staining, 400×); (**e**) Positive expression of CgA was observed in NET cells and neuroendocrine cells with background gastric mucosal hyperplasia (CgA immunostaining, 100×); (**f**) A well-differentiated G1 NET, with a Ki67 proliferation index of 1% (Ki67 immunostaining, 200×)

Among the 229 patients, 159 (69.4%) G1 tumors were found, whereas 60 (26.2%) G2 lesions and no G3 tumors were identified. The Ki-67 index ranged from 1 to 15%, and mitotic rates ranged from 0 to 8 per 2 mm^2^. Regarding the depth of tissue invasion, tumors were located in the mucosa in 89 (64.0%) of the 139 patients, tumors had invaded the submucosa in 47 patients (33.8%), and 3 tumors (2.2%) had infiltrated into the muscularis propria. The pathological reports of 172 patients had a description of the mucosal background, of which 168 cases (97.6%) involved marked atrophy of oxyntic glands and reduction in inherent glands, and 157 cases (91.3%) involved linear hyperplasia, micronodular hyperplasia or nodular hyperplasia of neuroendocrine cells. Ten patients out of 152 (6.6%) had histological evidence of *H. pylori* infection. Figure 2 shows the typical histopathology of a gastric NET with AIG.

#### Laboratory findings and 24-hour gastric pH monitoring

A total of 195/202 (96.5%) patients had elevated fasting serum gastrin levels. PCA was present in 181/222 (81.5%) patients, positive IFA was found in 56/126 (44.4%) patients, vitamin B12 deficiency was noted in 189/220 (85.9%) patients, decreased serum ferritin levels in 111/219 patients (50.7%), and decreased hemoglobin levels in 85/214 (39.7%) patients were reported. Abnormal thyroid function was detected in 58/203 (28.6%) patients, and 95/202 (47.0%) patients had positive thyroid antibodies (TPO-Ab and/or TG-Ab). The intragastric pH was significantly higher in 73/73 (100%) patients.

#### Tumor stages

Five (2.6%) of 195 patients with available staging information presented metastasis: 3 patients with regional lymph node metastasis, 1 patient with liver metastasis, and 1 patient with combined nodal and liver metastasis. One hundred sixty cases were classified as stage I, 30 as stage II, 3 as stage III, and 2 as stage IV.

#### Comorbidities

The most common comorbid diseases in the patients were anemia (39.7%) and Hashimoto’s thyroiditis (29.3%), and 6.4% of patients had other autoimmune diseases, including rheumatoid arthritis, Sjögren’s syndrome, vitiligo, and palmoplantar pustulosis. In addition, 9.0% of the patients had other malignant tumors, including colorectal cancer, gastric cancer, lung cancer, duodenal NETs, and rectal NETs. In terms of other endoscopic abnormalities, the main ones were hyperplastic polyps (16.6%), fundic gland polyps (3.9%), inflammatory polyps (3.1%), gastric xanthomatosis (2.6%), and gastric intraepithelial neoplasia (2.2%).

Clinicopathological findings at diagnosis of the 229 cases of type 1 gastric NETs are shown in Table 2.

**Table 2:** Clinicopathological characteristics of 229 patients with type 1 gastric NETs

Patient features	No. of patients	%
Sex		
Male	93	40.6%
Female	136	59.4%
Main symptoms		
Abdominal distension	59	25.8%
Abdominal pain	46	20.1%
Belching	21	9.2%
Postprandial fullness	20	8.7%
Dizziness	18	7.9%
Lower limb pain or numbness	5	2.2%
Other symptoms	68	29.7%
Asymptomatic	38	16.8%
Number of lesions		
Single	47	20.5%
Multiple	166	72.5%
Maximum diameter of tumor (cm)		
< 0.5	63	27.5%
0.5–0.9	89	38.9%
1.0–1.9	35	15.3%
≥ 2.0	9	3.9%
Unknown	33	14.4%
Tumor shape		
Polypoid	197	86.0%
Erosion or ulcer	6	2.6%
Coarse granular	5	2.2%
Polyp with erosion	12	5.3%
Unknown	9	3.9%
Depth of invasion (pathological)		
Mucosa	89	38.9%
Submucosa	47	20.5%
Muscularis propria	3	1.3%
Unknown	90	39.3%
Histological grade		
NET G1	159	69.4%
NET G2	60	26.2%
NET G3	0	0
Unknown	10	4.4%
Tumor stage		
Stage I	160	69.9%
Stage II	30	13.1%
Stage III	3	1.3%
Stage IV	2	0.9%
Unknown	34	14.8%
Comorbidities		
Anemia	85	39.7%
Hashimoto’s thyroiditis	58	29.3%
Other autoimmune diseases	9	6.4%
Other malignant tumors	12	9.0%

Data are expressed as numbers (percentages)

Abbreviations: NET, neuroendocrine tumor

### Treatment interventions

Of the 215 patients with treatment records, 207 had received one or more treatments for tumors. A total of 176 patients underwent endoscopic treatment, including 90 patients who underwent forceps biopsy, 85 patients who underwent endoscopic mucosal resection (EMR), 61 patients who underwent endoscopic submucosal dissection (ESD), 13 patients who underwent argon plasma coagulation (APC), and 2 patients who underwent cold snare polypectomy (CSP). Surgical procedures were performed in 15 patients, 3 of whom underwent radical total gastrectomy, 3 of whom underwent distal gastrectomy, 4 of whom underwent proximal gastrectomy, and 5 of whom underwent local tumor resection. Twenty-four patients were treated with somatostatin analogues (SSA), and 139 patients received traditional Chinese medicine (TCM) treatment for more than 6 months. Eight patients had noninterventional management based on endoscopic surveillance without initial resection.

All patients with treatment records had received treatments for comorbidities closely related to type 1 gastric NETs or AIG, such as pernicious anemia, iron-deficiency anemia, and peripheral neuropathy, by treatment with vitamin B12 supplementation and/or iron supplementation.

### Recurrence rate and clinical outcome

Gastroscopy follow-up data were available for 170 patients. Seventy patients (41.2%) experienced the first recurrence after a median follow-up of 31 months (range: 2-122 months), and the median RFS was 43 months. The 1-, 2-, and 3-year cumulative recurrence-free survival rates were 71.8%, 56.8%, and 50.3%, respectively. A total of 205 patients had follow-up survival data. During a median follow-up of 39 months (range: 2-132 months), 1 patient had bilateral pulmonary metastasis, and no patient had disease-related death.

### Risk factors for recurrence

**Fig. 3:**
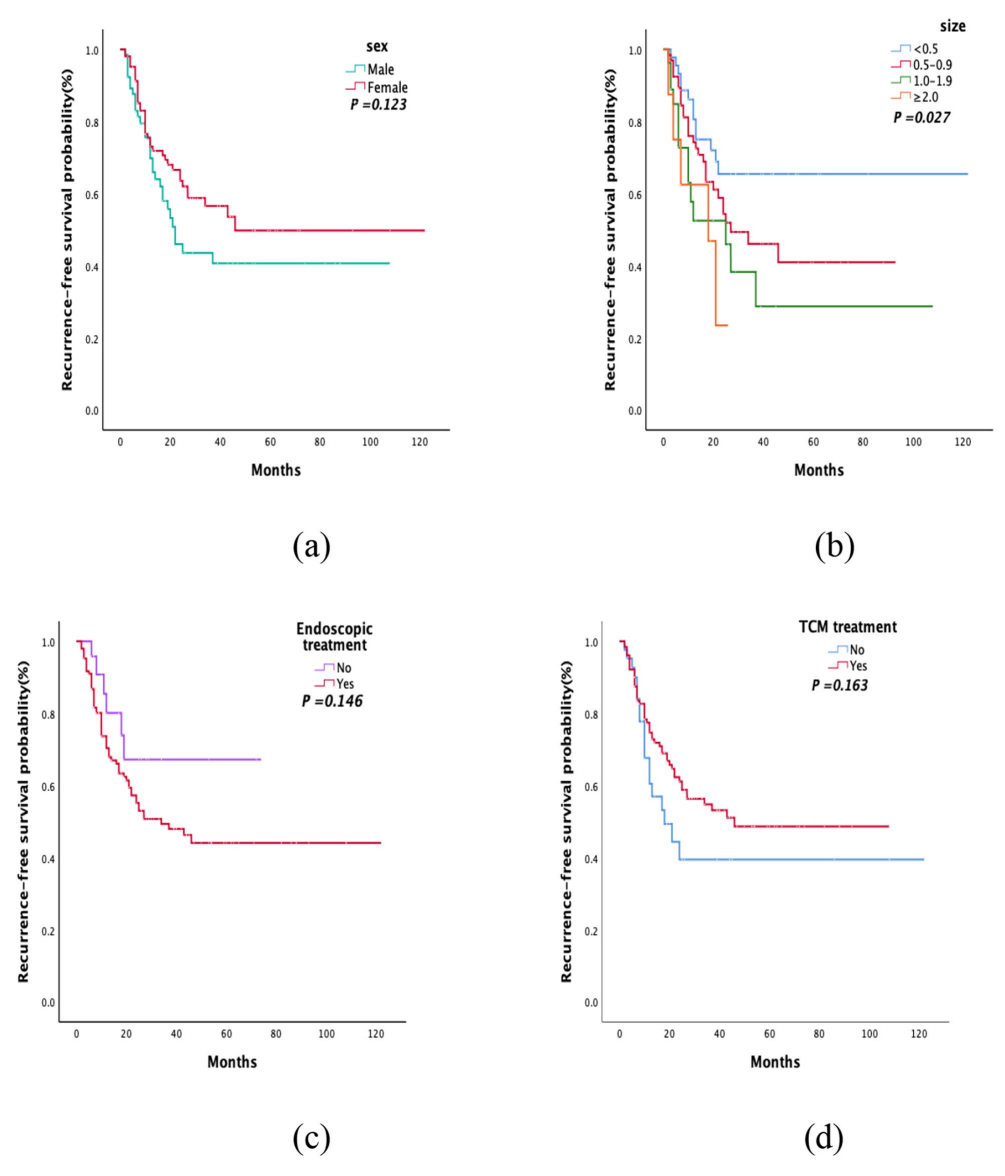
Recurrence-free survival curves of the patients. (**a**) K‒M curve of sex, (**b**) K‒M curve of tumor size, (**c**) K‒M curve of endoscopic treatment, (**d**) K‒M curve of TCM treatment

Tables 3 and 4 and Supplementary Table 1 show the analysis of possible risk factors for type 1 gastric NET recurrence using log-rank testing and the Cox proportional hazards regression model. As shown in Table 3, univariate regression analysis revealed that only tumor size had a statistically significant effect on recurrence (*P* < 0.05). However, although there were differences in the survival curves (Fig. 3) of sex, endoscopic treatment, and TCM treatment, they were not statistically significant. The multivariate regression analysis is shown in Table 4, and it suggested that in addition to tumor size, sex and whether the patient received TCM treatment were independent risk factors for recurrence (*P* < 0.05). Women had a lower risk of recurrence than men (HR = 0.53, 95% CI 0.32–0.88). Compared with patients with tumor sizes < 0.5 cm, the risk of recurrence was significantly increased in those with tumor sizes of 1 to 1.9 cm (HR = 2.72, 95% CI 1.27–5.81). Patients receiving TCM treatment had a lower risk of recurrence than patients who did not receive TCM treatment (HR = 0.55, 95% CI 0.31–0.99).

**Table 3:** Univariate analysis of possible risk factors for type 1 gastric NET recurrence

Factor	n/N	Median RFS (months)	*P*	HR (95% CI)*
Sex				
Male	31/65	22	0.123	1
Female	39/105	46		0.70 (0.43–1.11)
Maximum diameter of tumor (cm)				
< 0.5	13/45	> 122	0.027	1
0.5–0.9	30/66	27		1.71 (0.89–3.28)
1.0–1.9	14/27	25		2.63 (1.23–5.60)
≥ 2.0	5/8	18		3.38 (1.20–9.54)
Endoscopic treatment				
No	6/24	> 122	0.146	1
Yes	64/146	34		1.83 (0.79–4.23)
TCM treatment				
No	18/42	18	0.163	1
Yes	52/128	46		0.69 (0.4–1.18)

*HR (95% CI) in univariate analysis was performed with the Cox proportional hazards regression model

Data are shown as numbers

Abbreviations: NET, neuroendocrine tumor; TCM, traditional Chinese medicine; CI confidence interval

**Table 4:** Multivariate analysis of possible risk factors for type 1 gastric NET recurrence

Factor	HR (95% CI)	P*
Sex		
Male	1	
Female	0.53 (0.32–0.88)	0.014
Maximum diameter of tumor (cm)		
< 0.5	1	
0.5–0.9	1.59 (0.83–3.06)	0.163
1.0–1.9	2.72 (1.27–5.81)	0.010
≥ 2.0	2.65 (0.93–7.53)	0.068
Endoscopic treatment		
No	1	
Yes	2.47 (0.88–6.93)	0.085
TCM treatment		
No	1	
Yes	0.55 (0.31–0.99)	0.046

*Variables with a *P* value of < 0.20 in univariate analysis were retained in multivariate regression analysis (available case analysis)

Data are shown numerically

Abbreviations: TCM, traditional Chinese medicine; CI confidence interval

## Discussion

Type 1 gastric NETs are the most common subtype of gastric NETs and are mainly characterized by multiple, small polypoid lesions in the background of atrophic gastritis of the gastric fundus and/or body [6, 12, 13] that are well-differentiated and rarely metastasize but are prone to relapse [1, 14]. In this retrospective study of 229 patients with type 1 gastric NETs evaluated or treated at our center over the past 11 years, there were more women than men (59.4%: 40.6%), and the main tumor manifestations were multiple (72.5%), small (66.4% <1.0 cm) polypoid lesions and atrophic changes (73.4%) in the background mucosa, with mainly grade G1 pathology (69.4%), rare metastases (2.2%), and high recurrence (41.2%), similar to the results that have been previously reported in the literature.

With respect to treatment, 81.9% of the 215 patients with treatment records in this group had undergone endoscopic treatment, including forceps biopsy, EMR, ESD, etc., which was consistent with the recommendations in international guidelines. However, the indications for endoscopic intervention have been a controversial topic [12]. The 2012 version of the European Neuroendocrine Tumor Society (ENETS) guidelines [15] suggested that all lesions should be resected as much as possible, and the 2016 version of the updated guidelines [16] indicated that the minimal approach should be to resect tumors ≥ 10 mm to reduce the risk of metastasis. The latest ENETS 2023 guidance paper recommended that endoscopic resection should be proposed in type 1 gastric NETs larger than 1 cm [17]. A 10-year follow-up study by G. Esposito et al. found that type 1 gastric NETs < 5 mm could be followed up by noninterventional endoscopic surveillance [18]. A retrospective analysis showed that type 1 gastric NETs ≤ 10 mm were unlikely to develop clinically significant tumor progression and did not require resection in most cases, suggesting that endoscopic surveillance could be extended every 2–3 years [19]. In China, most doctors have always considered 1 cm to be too conservative as the cutoff for endoscopic resection; thus, tumors ≥ 5 mm in size have generally been recommended for resection, and 62.0% of patients receiving endoscopic treatment in our study had tumors ≥ 5 mm. Our analysis of risk factors for type 1 gastric NET recurrence also found that tumors < 5 mm have a significantly lower risk of recurrence or progression, which further supports the practice of only removing tumors ≥ 5 mm. However, with the deepening of research, our understanding of type 1 gastric NETs will continue to be updated.

Although type 1 gastric NETs are usually treated with an endoscopic approach, the ENETS 2023 guidance paper suggested that surgical resection is recommended in all tumors that are greater than 20 mm in size or involve suspected muscularis propria invasion. There were 15 patients in this study treated with surgical procedures, but 5 patients with tumors < 10 mm, no lymph node metastasis and no invasion of the muscularis propria also underwent partial gastrectomy, which also reflects that therapeutic measures were not standardized to a certain extent. This also highlights the importance of conducting a comprehensive evaluation of patients to avoid overtreatment. While considering the necessity of surgery, we should also pay special attention to potential postoperative complications and their impact on patient quality of life. In addition, 24 patients received SSA treatment, and approximately 80% of these patients could not undergo resection or had multiple larger tumors or frequent relapse. SSA therapy is associated with a high complete response rate, but relapse is frequently observed after discontinuation of therapy. Thus, continuous therapy would be the appropriate approach [17]. However, we also need to consider the potential side effects of long-term use, such as abdominal discomfort, cholelithiasis, diarrhea, fatigue, and weight loss [20]. Nevertheless, due to the limitations of retrospective studies and the lack of long-term follow-up for some patients, we were unable to systematically evaluate the adverse reaction profile of the patients. This is one of the limitations of this study.

Type 1 gastric NETs are a chronic disease related to AIG, which is often combined or complicated by certain diseases in the course of the disease, and therefore needs to be recognized as a systemic disease [21]. In addition to the presence of NET lesions in the stomach, non-NET lesions may also occur [21–24]. In this study, 25.3% of our patients had non-NET lesions, including hyperplastic polyps, fundic gland polyps, inflammatory polyps, and gastric xanthomatosis, suggesting that gastrin may play a trophic role on gastric mucosa [22], and we need to carefully distinguish these nonendocrine lesions from gastric NETs in clinical practice. Additionally, AIG is strongly associated with other autoimmune diseases [21]. An analysis showed that approximately two-thirds of AIG patients were diagnosed with at least one other autoimmune disease, the most common being Hashimoto’s thyroiditis and type 1 diabetes [23, 25]. A total of 29.3% of patients in this study had comorbid Hashimoto’s thyroiditis, and some patients had autoimmune diseases such as rheumatoid arthritis and Sjögren’s syndrome. Additionally, patients need to be vigilant and concerned about AIG-related complications, such as pernicious anemia, iron-deficiency anemia, and peripheral neuropathy [26], which are caused by vitamin B12 deficiency and iron absorption disorders [27]. In addition, we found that 13 patients had other malignant tumors, among which gastrointestinal tumors (66.7%) were the most common. A retrospective analysis revealed that 19.1% of 225 patients with NENs had other malignancies, and the most frequent cancers were prostate cancer, colorectal cancer, breast cancer, lung cancer, melanoma, and gastric cancer [28]. There have been few reports on the combination of type 1 gastric NETs with malignant tumors, and whether susceptibility genes play a role in the pathogenesis is still unknown. At present, we should focus on the overall consideration of our treatment to help prioritize decisions. Accordingly, attention should not only be given to the tumor and AIG itself but also to closely related conditions associated with other diseases, and they should be addressed in a timely manner, whether in the initial diagnosis or in the follow-up.

Type 1 gastric NETs are a well-recognized recurrent disease. The recurrence rate after endoscopic treatment is as high as 56.9–63.6% according to previous reports, and the median recurrence time is 8–39 months [18, 19, 29, 30]. After a median follow-up of 31 months, 41.2% of patients in our study experienced a first recurrence, and the median RFS was 43 months, which was slightly longer than that reported in the literature. Survival analysis revealed that the median RFS of patients who received TCM treatment (46 months) was significantly longer than that of patients who did not (18 months). Further multivariate analysis suggested that receiving TCM treatment could reduce the risk of recurrence. 61% of patients in our study had received TCM treatment, which may also be one of the reasons for the low overall recurrence rate and prolonged recurrence time of patients. A clinical controlled observation study also found that SMLJ01 (a Chinese herbal decoction) can help reduce the recurrence rate, relieve symptoms and prolong the recurrence time in patients with type 1 gastric NETs [31], which may be considered a viable adjuvant therapy option in the postendoscopic treatment of patients with type 1 gastric NETs. However, because most of our patients in this study received one or more treatments and important clinicopathological features could not be evaluated in some patients, it is relatively difficult to directly compare the impact of different treatment methods on recurrence, which can also affect the exploration and analysis of risk factors for recurrence. In addition, it was reported that proton pump inhibitors (PPIs) play a potential role in the occurrence and recurrence of gastric NETs and that PPIs may increase the risk of developing gastric NETs [32–34]. Unfortunately, we failed to collect information on the prior use of PPIs from all patients during their initial visit to our center, so the role of PPIs in type 1 gastric NETs was not evaluated in this study. Future multicenter randomized studies with large sample sizes and longer follow-up periods will be needed, and they may more comprehensively evaluate the effectiveness and safety of different therapies for preventing the recurrence of type 1 gastric NETs and enable the exploration of the risk factors for the onset and recurrence of type 1 gastric NETs.

## Conclusions

With the widespread application of endoscopic techniques and the improvement in public awareness of the disease in the past 30 years, the detection rate of type 1 gastric NETs has been increasing yearly. This study is probably the largest retrospective single-center study of patients with type 1 gastric NETs to date. It not only describes the clinicopathological features and treatment interventions of type 1 gastric NETs but also observes clinical outcomes and explores risk factors for recurrence. This retrospective study further indicates that type 1 gastric NETs have a high rate of recurrence, which underscores the importance of long-term and comprehensive management of type 1 gastric NETs. In clinical work, it is necessary not only to treat and perform follow-up of type 1 gastric NETs but also to strengthen the attention of related complications and associated diseases to achieve the best quality of life and survival prognosis.

### Electronic supplementary material

Below is the link to the electronic supplementary material.

Supplementary Table 1: Univariate analysis of possible risk factors for type 1 Gastric NET recurrence

## Data Availability

All data generated or analyzed during this study are included in this published article.
