# Supplementary material for: Management of type 1 gastric neuroendocrine tumors: an 11-year retrospective single-center study
Source: BMC Gastroenterol. 2023 Dec 14;23:440. doi: 10.1186/s12876-023-03079-6 (PMC10722838; doi:10.1186/s12876-023-03079-6)
Supplement: Supplementary file 1 — Supplementary Table 1: Univariate analysis of possible risk factors for type 1 Gastric NET recurrence [file 12876_2023_3079_MOESM1_ESM.docx]

**Supplementary Table 1.** Univariate analysis of possible risk factors for type 1 Gastric NET recurrence

| Factor | n/N | Median RFS (months) | *P* | HR (95% CI) *** |  |
| --- | --- | --- | --- | --- | --- |
| Age at diagnosis (years) |  |  |  |  |  |
| <50 | 32/78 | 37 | 0.994 | 1 |  |
| ≥50 | 38/92 | 43 |  | 1.00 (0.63-1.60) | |
| Number of lesions |  |  |  |  |  |
| Single | 15/35 | 22 | 0.757 | 1 |  |
| Multiple | 53/122 | 37 |  | 0.92 (0.52-1.63) | |
| Depth of invasion (pathological) |  |  |  |  |  |
| Mucosa | 27/72 | >122 | 0.257 | 1 |  |
| Submucosa | 20/42 | 24 |  | 1.45 (0.81-2.60) | |
| Muscularis propria | 0/2 | - |  | - |  |
| Histological grade |  |  |  |  |  |
| NETG1 | 47/115 | 27 | 0.917 | 1 |  |
| NETG2 | 22/48 | 37 |  | 0.97 (0.57-1.62) | |
| Tumor stage |  |  |  |  |  |
| Stage I | 47/116 | 46 | 0.287 | 1 |  |
| Stage II | 13/26 | 25 |  | 1.58 (0.85-2.92) | |
| Stage III/IV | 2/3 | 18 |  | 1.62 (0.39-6.67) | |
| Surgical procedures |  |  |  |  |  |
| No | 64/159 | 46 | 0.294 | 1 |  |
| Yes | 6/11 | 18 |  | 1.55 (0.67-3.59) | |
